# Supplementary figures and images for: Dexamethasone is associated with early deaths in light chain amyloidosis patients with severe cardiac involvement
Source: PLoS One. 2021 Sep 15;16(9):e0257189. doi: 10.1371/journal.pone.0257189 (PMC8443042; doi:10.1371/journal.pone.0257189)

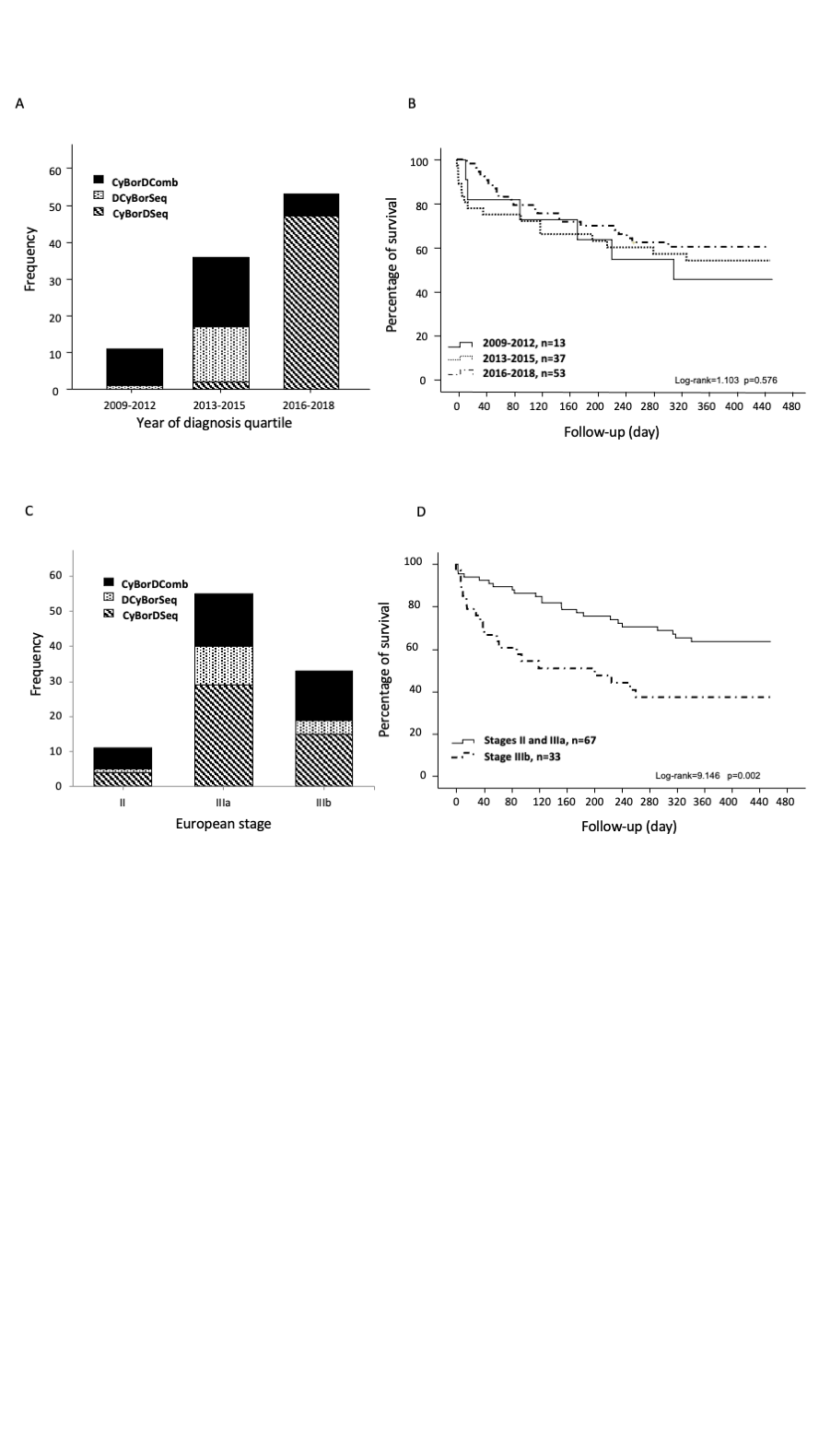

Supplement: S1 Fig — (A) Chemotherapy regimens used in treated patients by year of diagnosis. (B) Survival by year of diagnosis. (C) Chemotherapy regimens used in treated patients by European stages. (D) Survival by European stage. (TIFF) [file pone.0257189.s001.tiff]
