# Supplementary material for: Dexamethasone is associated with early deaths in light chain amyloidosis patients with severe cardiac involvement
Source: PLoS One. 2021 Sep 15;16(9):e0257189. doi: 10.1371/journal.pone.0257189 (PMC8443042; doi:10.1371/journal.pone.0257189)
Supplement: S1 Table — (PDF) [file pone.0257189.s002.pdf]

Supplementary file 2: characteristics and description of cumulative dose of dexamethasone administered to patients who died during the first cycle of chemotherapy

Group of chemotherapy	Patient Number	Delay between first dose of dexamethasone and death (days)	Cumulative dose of dexamethasone (mg)	European score	Baseline NTproBNP pg/ml	Baseline Troponin T HS ng/ml
CyBorDComb	1	0	20	IIIb	9590	173
	2	2	20	IIIa	1979	66
	3	3	160	IIIa	6938	104
	4	10	80	IIIb	43071	132
	5	11	40	IIIa	2372	171
	6	15	80	IIIb	20661	348
	7	16	60	IIIb	32909	180
DCyBorSeq	8	2	40	IIIa	4000	150
	9	7	20	IIIb	29035	133
	10	7	160	IIIb	33638	NA
	11	8	120	IIIb	109857	290
CyBorDSeq	12	29	60	IIIb	16463	126
