# Supplementary material for: Dexamethasone is associated with early deaths in light chain amyloidosis patients with severe cardiac involvement
Source: PLoS One. 2021 Sep 15;16(9):e0257189. doi: 10.1371/journal.pone.0257189 (PMC8443042; doi:10.1371/journal.pone.0257189)
Supplement: Attachment — Submitted filename: Response to Editor.docx [file pone.0257189.s003.docx]

August 10^th^, 2021

Mrs. Mélanie Bézard

Department of Cardiology

Henri Mondor Hospital

51 avenue du Marechal de Lattre de Tassigny

94000 Créteil, France

Doctor Emily Chenette

Editor-in-Chief

Plos One

Dear Doctor Chenette,

We thank you for your interest in our work and for the time you have devoted to us.

Following your advice, we have listed each of the Editor comments with our response being added underneath each one. We have added an exact copy of the revised text from the manuscript (where appropriate) beneath each response.

Hoping that these answers give you complete satisfaction,

Yours sincerely

Mélanie Bézard

Corresponding author

**Response to editor**

1. Please amend your current ethics statement to include the full name of the ethics committee/institutional review board(s) that approved your specific study.

**Response**: the study was approved by the French data protection authority: Comité National de l’Informatique et des Libertés, CNIL, #1431858.

L101-103, we added the sentence: “All patients gave informed consent for the use of their anonymized data. The study complied with the Declaration of Helsinki and was approved by the French data protection authority (Comité National de l’Informatique et des Libertés, CNIL, #1431858).”

2. We note your current data availability statement:

"The data cannot be shared publicly due to the information it contains about patients. Some data is available upon request."

Before we proceed, we’ll require some additional information to ensure your submission adheres to the PLOS policy on acceptable data access restrictions: <https://journals.plos.org/plosone/s/data-availability#loc-acceptable-data-access-restrictions>.

1) Please provide all necessary instructions and non-author contact information (preferably email) for a data access committee, ethics committee, or other institutional body other researchers would require to apply for data access. Note that it is not acceptable for an author to be the sole named individual responsible for ensuring data access.

**Response:** Data can be shared upon request from ARMDC (Association pour la recherche multi-disciplinaire en cardiologie), Hôpital Henri Mondor, Service de cardiologie, unité insuffisance cardiaque et amylose, 51 avenue du maréchal de Lattre de Tassigny, 94000 Créteil, thibaud.damy@aphp.fr
